# Factors Influencing the Duration of Urine Leakage following Percutaneous Nephrolithotomy

**DOI:** 10.1155/2014/105709

**Published:** 2014-02-04

**Authors:** Ugur Uyeturk, Adnan Gucuk, Eray Kemahli, Emine Dagistan, Mevlut Yildiz, Burak Yilmaz, Ahmet Metin

**Affiliations:** ^1^Department of Urology, Abant Izzet Baysal Medical Faculty, 14280 Bolu, Turkey; ^2^Department of Radiology, Abant Izzet Baysal Medical Faculty, 14280 Bolu, Turkey

## Abstract

*Purpose.* The duration of urine leakage following the removal of the nephrostomy tube after percutaneous nephrolithotomy (PCNL) shows significant variations depending on the techniques used. We aimed to assess the factors likely to influence the duration of urine leakage. *Material and Methods.* In total, 103 patients who underwent PCNL were reviewed retrospectively. DUL was evaluated regarding patient characteristics, thickness of the access line, presence of hydronephrosis, and residual stones. *Results.* DUL was significantly prolonged in accordance with a decrease in the thickness of parenchyma tissue (*R* = −0.716, *P* < 0.001). DUL was prolonged as the degree of hydronephrosis (*R* = 0.526, *P* < 0.001) and the number of patients with residual stones (*R* = 0.273, *P* = 0.005) increased. Median DUL was significantly longer in patients with residual stones than those without residual stones (*P* = 0.002). In the receiving operating curve analysis, the optimum cut-off value of parenchymal thickness for hospitalization ≤12 h was 17.2 mm (sensitivity, 90.2%; specificity, 69.4%; *P* = 0.001). *Conclusions.* We found that parenchymal thickness of the access line, hydronephrosis, and residual stones were the most influential factors determining DUL following PCNL, respectively.

## 1. Introduction

Percutaneous nephrolithotomy (PCNL), which was first performed in the late 1970s, heralded a breakthrough in the treatment of renal stones [1]. It has remained the preferred technique and has become the gold standard for large (>2 cm) or stag-horn stones that are difficult to treat [2, 3]. The postoperative success rate with PCNL is as high as with open surgery, and it is associated with a shorter hospital stay and fewer complications than open surgery [4].

Major complications of the procedure include ileus, pneumonia, deep-vein thrombosis, excessive bleeding requiring embolization, and hydrothorax. Persistent urine leakage, nephrostomy tube slippage, and urinary system infections are minor complications of PCNL [5]. The duration of urine leakage (DUL) following removal of the nephrostomy tube after PCNL varies significantly depending on the techniques used. Prolonged DUL has the potential to extend hospital stay [6]. Few studies have evaluated the relationship between prolonged DUL and the demographic and clinical characteristics of patients. In the present study, we assessed the factors which are likely to influence DUL following PCNL.

## 2. Materials and Methods

**Figure 1:**
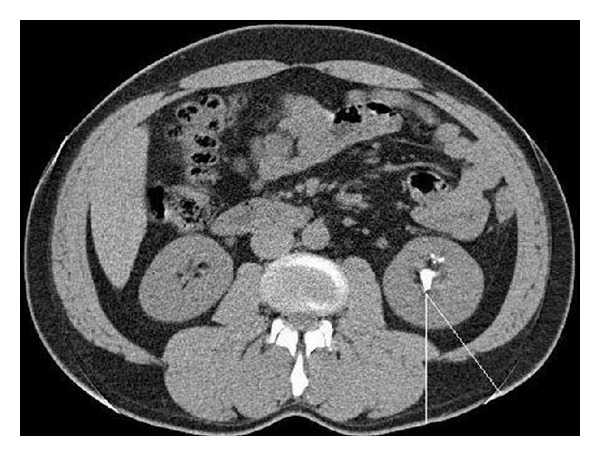
Thickness of the subcutaneous adipose tissue, muscle tissue, visceral adipose tissue, and parenchyma tissue at the percutaneous access site was determined by measuring the access line between the calyx that underwent percutaneous nephrolithotomy and skin at a 30° angle to a vertical line.

In total, 103 patients who underwent PCNL between February 2011 and March 2013 in the Urology Department of our hospital were retrospectively evaluated for urine leakage following PCNL after receiving approval from the ethics committee. Prior to the procedure, all patients underwent urinalysis and bacteriological analyses, a complete blood count, serum biochemistry, and coagulation tests. Hydronephrosis grades 0–4 were assessed by various imaging methods, including ultrasonography (USG), intravenous pyelography, or computed tomography (CT), as described previously [7]. Preoperative CT was used to measure the thickness of the tissue in the access line and the volume of the stones. Hounsfield unit density was calculated to determine urinary stone composition, as described previously [8]. The volume of the stone was calculated according to guidelines established by the European Association of Urology [9]. The thickness of tissue in the percutaneous access line was determined by separately measuring the thickness of the subcutaneous adipose tissue, the muscle tissue, the visceral adipose tissue, and the parenchyma tissue in the access line between the calyx that underwent PCNL and the skin at a 30° angle to the vertical line (Figure 1). All radiological measurements were performed by a single radiologist and the urologists who performed the procedure.

Our surgical PCNL technique was described previously [10]. Briefly, following administration of general anesthesia, a 6-F open-ended ureteral catheter was placed in the patient in the lithotomy position to view the anatomy of the collecting system. Next, the patient was returned to the prone position, and the collecting system was viewed via fluoroscopy using a radiocontrast medium. Twenty-six-F Amplatz dilators (Microvasive/Boston Scientific, Natick, MA, USA) were used to dilate the tract, and a 26-F Amplatz sheath was inserted. Next, only a 24-F rigid nephroscope, pneumatic lithotripter, and grasping forceps were used. Fragments accessible via rigid nephroscopy were cleared with grasping forceps. Collecting system intactness was evaluated via fluoroscopic imaging and antegrade nephrostography intraoperatively. The procedure was completed by placing a 14-F nephrostomy tube into the calyx. However, ureteral stents were not placed at the time of surgery. The surgical duration was calculated as the time from insertion of the ureteral catheter and placing the patient in the prone position to insertion of the nephrostomy tube.

An appropriate dose of prophylactic cephalosporin was given to the patients prior to anesthesia. Antibiotic therapy was continued until removal of the nephrostomy tube. Bladder catheters were removed from the patients on postoperative day 1. The decision about removing the nephrostomy tube was based on the color of the urine coming from the tube and normal antegrade nephrostogram findings. The percutaneous access site dressing was replaced when the patient informed the physician that the dressing had become wet. Furthermore, dressings of all patients were changed at 8 h intervals. DUL was the time between removing the nephrostomy tube and the last wet dressing. Urine leakage persisting 48 h after removal of the nephrostomy tube was considered “prolonged urine leakage,” and a 4.7-F double-J-stent was placed in these patients [11].

Residual stones were assessed postoperatively by direct urinary systemgraphy, USG, or CT scan. The procedure was considered successful if the patient was either stone free or had only a clinically insignificant residual fragment (residue < 4 mm). Larger stones were considered residual stones and were evaluated. The mean decrease in hematocrit was calculated, considering the hemograms that were obtained 24 h before surgery and 36 h after surgery, along with any blood transfusions.

Patients who had undergone multiple accesses or re-PCNL without removal of the nephrostomy tube, patients with a postoperative body temperature >38°C despite antibiotic prophylaxis, and patients with preoperative urethral catheter were not included in the study.

## 3. Statistical Analyses

The data were analyzed using the SPSS for Windows 11.5 software. The Shapiro-Wilk test was used to determine whether the distribution of continuous variables was normal. The descriptive statistics are presented as means ± standard deviation or as medians (range) for continuous variables and as the numbers of cases for categorical variables.

Significance of the median values between the groups was analyzed with the Mann-Whitney *U* test. The presence of a significant correlation between the continuous variables was investigated using Spearman's correlation test. The capacity of parenchymal thickness for predicting the presence of hospitalization ≤12 h in patients with urine leakage after PCNL was analyzed using receiver operating characteristic curve analysis. Sensitivity and specificity are presented when a significant cut-off value was observed. A value of *P* < 0.05 was taken to indicate statistical significance.

## 4. Results

The patients had a median age of 50.4 years (range: 17–80 years), and 36 (35%) were female and 67 (65%) were male. Sixteen (15.5%) patients had a history of extracorporeal shock wave lithotripsy (SWL), and seven (6.8%) had a history of kidney surgery. Hydronephrosis was not present in 23 (22.3%) patients, but 42 (40.8%) had grade 1, 13 (12.6%) had grade 2, 21 (20.4%) had grade 3, and four (3.9%) had grade 4 hydronephrosis. The median surgery duration was 85 min (range: 45–185 min). The mean decrease in postoperative hematocrit was 4.3 ± 2.7% (Table 1).

The median stone volume value was 4608 mm^3^ (range: 636–17.688 mm^3^). Mean renal parenchymal thickness in the percutaneous access line was 17.2 ± 7.2 mm, median visceral adipose tissue thickness was 15 mm (range, 2.8–35.3 mm), median muscle tissue thickness was 12.1 mm (range, 2.5–78.2 mm), and mean subcutaneous tissue thickness was 26 ± 11.4 mm. Median DUL through the access site after removal of the tube was 12 h (range, 3–51 h). A residual stone was detected after the procedure in 16 (15.5%) patients (Table 2).

**Figure 2:**
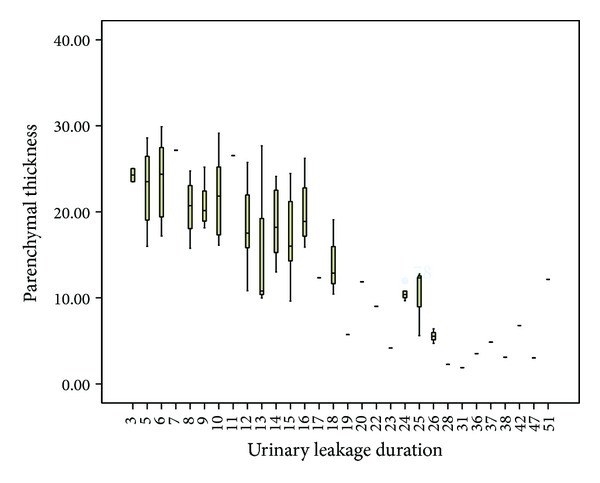
A positive correlation between the parenchymal thickness and the duration of urine leakage duration.

No significant relationship was observed between DUL and age (*P* = 0.888), surgery duration (*P* = 0.529), amount of bleeding (*P* = 0.302), or stone volume (*P* = 0.082). However, DUL was prolonged as the degree of hydronephrosis (*R* = 0.526, *P* < 0.001) and the presence of residual stones (*R* = 0.273, *P* = 0.005) increased. No significant relationship was found between DUL and the thickness of the visceral adipose tissue (*P* = 0.707), muscle tissue (*P* = 0.882), or subcutaneous adipose tissue at the access site (*P* = 0.226). However, DUL was significantly prolonged in accordance with a decrease in the thickness of the parenchyma tissue (*R* = −0.716, *P* < 0.001) (Figure 2) (Table 3).

**Figure 3:**
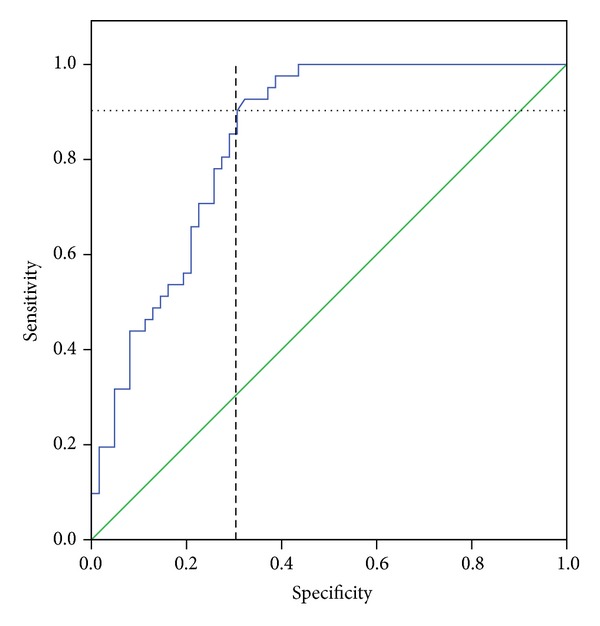
Receiving operating curve analyses for the optimum cut-off value of parenchymal thickness in hospitalizations ≤12 h.

Median DUL was 17 h (range: 3–47 h) in patients with residual stones and 11 h (range: 3–51 h) in those without residual stones. A significant difference in median DUL was observed in patients with or without residual stones (*P* = 0.002). The optimum cut-off value of parenchymal thickness for hospitalization ≤12 h was 17.2 mm (sensitivity, 90.2%; specificity, 69.4%; positive predictive value, 66.1%; negative predictive value, 91.5%; accuracy, 78.64%; area under the curve, 0.842; *P* < 0.001; upper band, 0.915; lower band, 0.768) (Figure 3).

## 5. Discussion

PCNL has long been the preferred method to treat stones >2 cm, stag-horn stones, and stones of the inferior lobe <1 cm. In addition to the high success rate, a low morbidity rate makes it more advantageous than other surgical techniques [4, 12]. A nephrostomy tube is placed into the nephrostomy tract after removing the stone during conventional techniques. This tube helps hemostasis and hinders extravasation of urine, thus, preventing the development of urinoma [13]. However, urine leakage from the drain site after removal of the postoperative nephrostomy tube is a common problem [14]. In a study performed using the Clavien grading system, urine leakage from the flank for <12 h was considered a grade 2 complication, whereas implementation of a double-J-stent due to urine leakage lasting longer than 24 h was considered a grade 3a complication. Urine leakage that prolongs hospital stay is the most common type of group 3a complication [15]. In another study, urine leakage lasting >1 week was detected in 1.5% of cases that underwent PCNL [16]. Prolonged complication duration leads to delayed discharge from hospital. In our study, we did not observe urinary leakage >1 week because persistent urine leakage after 48 h was managed with a double-J-stent.

In a study of post-PCNL complications, urine leakage lasting <24 h was detected in 15% of patients [17]. However, it was concluded that ignorance and/or inadequate data records might have caused an underestimation of minor complications in that retrospective study. This complication was followed by body temperature >38°C, which was recorded at least once in 11% of patients, and bleeding requiring transfusion, which was seen in 6.9% of patients. Fever could be encountered more frequently in patients with stag-horn stones and bleeding might decrease along with duration of the PCNL surgery [17]. The most common complications reported in another study included blood transfusion (10.9%), double-J-stent implantation due to urinary leakage lasting >24 h (4.6%), and urinary leakage lasting <12 h (4%). The need for a blood transfusion may increase during treatment of complex stones [15]. In the present study, no relationship was found between the postoperative decrease in hematocrit level and DUL.

In a study of the relationship between the duration of postoperative hospital stay and patient age, age had no effect on hospital stay duration [18]. Another study including patients >60 years and <60 years reached the same conclusion [19]. We also concluded that age did not influence DUL.

One study evaluated the relationship between the degree of renal hydronephrosis and DUL. That study concluded that duration increases in line with the degree of hydronephrosis [20]. The present study reached the same conclusion. However, parenchymal thickness in the access line was more significant for DUL than the degree of hydronephrosis. The reason may be the uncertainty in the division of hydronephrosis into five grades and not being able to describe the local parenchyma impaired by the observer, whereas access to parenchymal thickness measurement is more sensible and scalable, and a particularly impaired local parenchyma related to a stone might be evaluated. We believe we can obtain a cut-off value for parenchymal tissue thickness by collecting numerical data. No other study of the effect of parenchymal thickness in the access line on DUL has been conducted. In our study, the optimum cut-off value of parenchymal thickness for hospitalization ≤12 h was 17.2 mm.

Some studies have demonstrated that stone load prolongs the duration of hospital stay [18, 21], whereas others have suggested the opposite [20, 22]. We concluded that stone load does not influence DUL.

Studies evaluating the effect of body mass index (BMI) on post-PCNL complications have reported different results. One study reported that BMI increases complications [23], but another found no relationship between BMI and complications [24]. We also found no relationship between subcutaneous adipose tissue thickness, which is an indirect measure of BMI, and DUL.

One study demonstrated that post-PNCL residual stone fragments increase DUL [25]. Similarly, DUL increased in line with the increase in residual stones in the present study. A study investigating the efficacy of PCNL in patients with a history of kidney surgery reported that past surgery prolongs the duration of PCNL but has no effect on success or morbidity [26]. Another study, including patients who had undergone SWL and open surgery in the past, demonstrated that previous procedures do not change either the success or the complication rates [27].

The results of the present study reveal that DUL was significantly associated with the degree of hydronephrosis in the relevant kidney, the parenchymal thickness of the access site, and the presence of the residual stone. We found that the thickness of the subcutaneous adipose tissue, muscle tissue, and visceral adipose tissue had no effect on DUL. However, parenchymal thickness in the access line influenced DUL.

## 6. Conclusions

This is the first study to report that parenchymal thickness in the access line was most significantly associated with DUL. Further studies are necessary to confirm our results in a greater number of patients.

## Figures and Tables

**Table 1:** Demographic and clinical characteristics of the PCNL patients were summarized (*n* = 103).

	*n* (%)
Gender	
Female	36 (35)
Male	67 (65)
Age (year)	50.4 (17–80)
SWL history	16 (15.5)
Surgery history	7 (6.8)
Kidney	
Left	41 (39.8)
Right	62 (60.2)
Grade of hydronephrosis	
0	23 (22.3)
1	42 (40.8)
2	13 (12.6)
3	21 (20.4)
4	4 (3.9)
Duration of the surgery (minute )	85 (45–185)

**Table 2:** The stones and clinical parameters.

The stone volume (mm^3^)	4608 (636–17688)
Renal parenchymal thickness in the access line (mm)	17.2 ± 7.2
Visceral adipose tissue thickness in the access line (mm)	15 (2.8–35.3)
Muscle tissue thickness in the access line (mm)	12.1 (2.5–78.2)
Subcutaneous tissue thickness in the access line (mm)	26 ± 11.4
Calix of puncture (*n*)	
Inferior	98 (95.1%)
Median	4 (3.9%)
Superior	1 (0.9%)
The duration of urine leakage (h)	12 (3–51)
Patients with residual stone (*n*)	16 (15.5%)

**Table 3:** The correlation between clinical parameters and the duration of urine leakage.

Clinical parameters	Correlation	*P* ^ a^
Age	−0.014	0.888
Duration of the surgery	0.063	0.529
Reduction in hematocrit level	0.103	0.302
Grade of hydronephrosis	0.526	<0.001*
The stone volume	0.172	0.082
Renal parenchymal thickness in the access line	−0.716	<0.001*
Visceral adipose tissue thickness in the access line	−0.038	0.707
Muscle tissue thickness in the access line	0.015	0.882
Subcutaneous tissue thickness in the access line	0.120	0.226
Patients with the residual stone	0.273	0.005*

^a^Spearman's correlation test; *statistically significant correlation.
